# Patricia Goldman-Rakic: a pioneer and leader in frontal lobe research

**DOI:** 10.3389/fnhum.2023.1334264

**Published:** 2024-01-29

**Authors:** Bryan Kolb

**Affiliations:** ^1^University of Lethbridge, Lethbridge, AB, Canada; ^2^Department of Neuroscience, University of Lethbridge, Lethbridge, AB, Canada

**Keywords:** working memory, brain plasticity, prefrontal cortex, brain development, dopamine, schizophrenia

## Abstract

Our understanding of the organization of the frontal cortex can be traced back to the experimental studies in the late 1800s by Fritsch and Hitzig on the frontal cortex of dogs and the frontal cortex of monkeys by Ferrier. These studies and many other studies that followed focused on motor functions, but halfway through the 20th century, very little was understood about the role of the frontal lobe in the control of other functions, and it was generally thought that the frontal lobe did not play a significant role in cognition. One result was that studies of cortical functions in cognition were carried out largely on parietal and temporal cortical regions with surprisingly little interest in the frontal lobe. The first systematic studies of the effects of prefrontal lesions on non-human primates began around 1950, especially by Rosvold and Mishkin in the Laboratory of Psychology at the National Institute of Mental Health (NIMH) in the United States. With her background in development, Pat Goldman joined this laboratory in 1965 and began an examination of the effects of prefrontal lobectomy on behavior in infant rhesus monkeys, both during development and later as the animals grew into adulthood. Her developmental studies were groundbreaking as they demonstrated that the effects of early prefrontal lesions varied with precise age (including prenatal), precise lesion location, behaviors measured, and age at assessment. She also began in parallel extensive studies of the role of the prefrontal cortex for a range of functions (especially working memory) in adult monkeys, which led to an examination of factors that influenced functional outcomes after injury or disease. This research was critical in helping to identify the significant role of the prefrontal cortex in cognition in both normal brains and neurological diseases such as schizophrenia. Her pioneering study demonstrating the role of the prefrontal cortex in cognition led to a remarkable increase in the number of researchers studying prefrontal functions in both non-human primates and rodents. This review will chronicle the key findings in her 35^+^ years studying the prefrontal cortex and illustrate the course she set for generations to follow.

## Introduction

Our understanding of frontal lobe function began with the classic studies of the organization of the motor cortex of dogs in the 1880s by Fritsch and Hitzig and the frontal cortex of monkeys by Ferrier. Although Jacobsen (1935, 1936) first demonstrated that disturbance of the frontal lobes in primates produced severe and permanent deficits on delayed-response-type tests and identified the dorsolateral prefrontal cortex (dlPFC) as the key region, the reliability of his finding went largely unstudied for over 20 years. There was a strong interest in frontal lobotomy as a psychotherapeutic procedure in the late 1940s and the 1950s, but this interest was focused on clinical issues and not on an understanding of the role of the frontal lobe in cognitive functions. Indeed, there was a strong view that the frontal lobe did not play a significant role in cognition (e.g., Hebb, 1945; Teuber, 1964). The first systematic studies of the effects of prefrontal lesions on non-human primates began around 1950, especially by Rosvold and Mishkin in the Laboratory of Psychology at NIMH in the United States. Several other laboratories began studies on the prefrontal cortex of monkeys, dogs, and cats in the 1950s, leading an important symposium on the “Frontal Granular Cortex and Behavior” in 1962. Presentations at this symposium summarized research on the prefrontal cortex of human, non-human primate, carnivore, and rodent brains. The subsequent volume (Warren and Akert, 1964) served as a major stimulus for expanding comparative studies on the prefrontal cortex and behavior as was revealed in a subsequent symposium and volume published in *Acta Neurobiolgiae Experimentalis* in 1972.

This volume, which included Patricia Goldman, was followed by an explosion of research on the prefrontal cortex and cognitive behavior, in which Pat played a leading role.

## Pat Goldman-Rakic’s early research biography

Pat obtained a Bachelor’s degree in Experimental Psychology in 1959 at Vassar College, followed by a PhD in Developmental Psychology at UCLA in 1963 with Wendell Jeffrey, a leading developmental psychologist at the time. This was followed by postdoctoral work at UCLA and the Museum of Natural History with a leading comparative psychologist Ethel Tobach. Her earliest publications (Goldman, 1965; Goldman and Tobach, 1967) reflected her developmental interest in behavior. Pat’s neuroscience career began when she joined H.E. Rosvold’s Section of Neuropsychology at the National Institute of Mental Health (NIMH) in 1965. There she learned how to perform brain lesions on monkeys, and her first publications were on the effects of prefrontal lobectomy in infant monkeys (Goldman et al., 1970a,b). These studies illustrated her continuing interest in development and her new interest in the prefrontal cortex (see also reviews by Arnsten, 2013, 2023). She married Pasko Rakic in 1979, and they both moved to Yale. Her new laboratory flourished as it produced monumental changes in our understanding of the frontal lobe and behavior. Unfortunately, Pat’s life was cut short when she was struck by a car while crossing a street in Hamden, Connecticut. She died 2 days later on 31 July 2003.

## Effects of early brain injury

Pat’s studies on the effects of early frontal lobe injury radically changed our understanding of the effects of early cortical injury. Beginning in the late 1930s, Margaret Kennard began to study the effects of motor cortex injury in infant monkeys and she reported that infant monkeys appeared to have a better functional outcome than adult monkeys with the same injuries (Kennard, 1942). Hans-Lukas Teuber called this phenomenon the *Kennard Principle*, by which he meant that if you are going to have a brain injury, have it early (Teuber, 1975). This conclusion had intuitive appeal because it is a common observation that infants seem to recover more quickly than adults from many maladies, and it was known that it is rare for children to have lasting aphasia even when language areas in the left hemisphere were severely compromised. The idea that earlier is better was tested in monkeys given dorsolateral prefrontal (dlPFC) lesions at 5 days of age by Akert et al. (1960) who found that in contrast to monkeys with lesions in adulthood, the monkeys with infant lesions could learn a delayed-response task as well as controls (for details on this task, see below in the discussion of working memory). This result was confirmed in a series of other studies including one by Pat (Harlow et al., 1964, 1968; Kling and Tucker, 1967; Goldman et al., 1971). Two proposed explanations for this result were (1) that anomalous neural connections formed after the early lesions, so the brain was able to recruit other regions to solve behavioral tasks normally controlled by the missing tissue (e.g., Hicks and D’Amato, 1970; Schneider, 1973) or (2) that other cortical regions compensated by committing to take over the missing prefrontal functions without a need to rewire the region.

Given that both the dlPFC and orbitofrontal (OFC) cortical regions received projections from the dorsal medial thalamic nucleus (MD) (Akert, 1964), the OFC was an obvious candidate for either possibility. Pat elected to use a test of object reversals to examine OFC function after infant lesions given that adult OFC operations are impaired on this task (Goldman et al., 1970a).

Goldman et al. (1970a,b) found that although monkeys with combined dlPFC and OFC in infancy were not impaired at tests of dlPFC function when the animals were studied at 1 year of age, the monkeys were as impaired on object reversals as were monkeys operated later in life. Thus, the Kennard Principle appeared to have exceptions. Indeed, over the next few years, evidence accumulated that early lesions in the motor or visual cortex did not provide significant benefits relative to similar adult lesions (e.g., Bland and Cooper, 1969; Doty, 1971). So, why was the effect of dlPFC lesions different?

**Figure 1:**
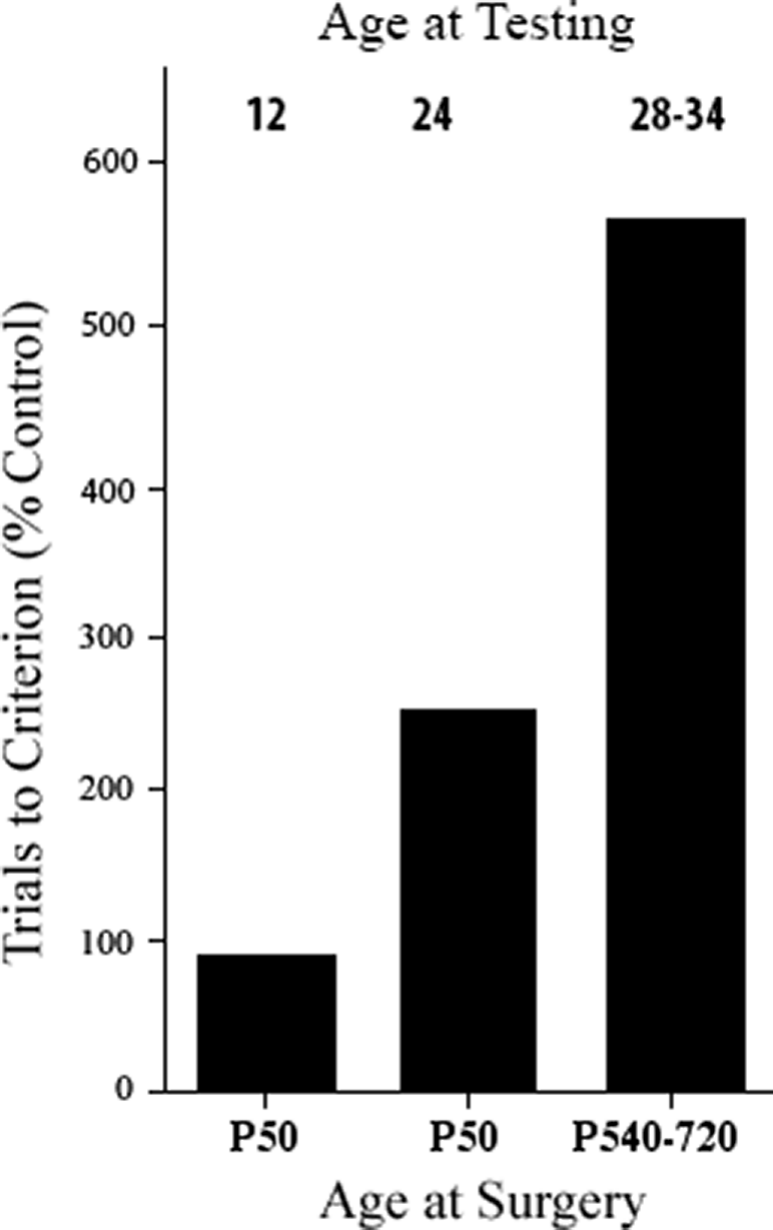
Effects of early and late dorsolateral prefrontal lesions on delayed response performance at specified times post-surgery. Ages are in months. All groups are statistically different from one another (based on Goldman, 1974).

One possibility was that the age at which the animals were tested behaviorally was important. For example, Kennard (1942) found sparing of motor functions when the monkeys with motor cortex lesions were tested early in life but noted that they appeared to begin to show deficits as they grew older. Similarly, although Tucker and Kling had shown good performance on delayed response after infant dlPFC lesions when they were tested at 10 months, one monkey tested again at 18 months showed severe impairment. This result was confirmed by Pat with a group of four monkeys with dlPFC lesions that performed as well as controls at 12 months; however, at 24 months, two monkeys had large impairments and two did not, and at 28 months, all four monkeys were severely impaired when tested (Goldman, 1972, 1974) (see Figure 1). Thus, it appeared that the dlPFC matured more slowly than the OFC. The reason that the dlPFC lesion animals could perform normally at the task was likely because other cortical regions such as the OFC and subcortical regions, such as the caudate nucleus, could solve the task, and the immature dlPFC was not yet involved. As the dlPFC matured, the functions would normally be taken over by this region, but in its absence, functional deficits would appear [see also Pat’s studies of cooling prefrontal regions in the developing monkey brain that also showed a similar result Goldman and Alexander, 1977; Alexander and Goldman, 1978]. Given this logic, one might ask whether the same might be true of the OFC if the functions were tested early enough. Indeed, Pat showed this to be the case—if monkeys with OFC lesions in infancy were tested behaviorally at 2.5 months of age, they performed as well as control animals but by 1 year they were impaired at the same tasks. Pat hypothesized that in the first few months, the caudate nucleus could solve the tasks as it was already mature shortly after birth, but this function was at least partly shifted to other cortical regions as they matured (Goldman, 1974). Similar conclusions can also be seen in children with early cortical lesions (Banich et al., 1990), and hamsters with early medial frontal lesions (Kolb and Whishaw, 1985).

**Figure 2:**
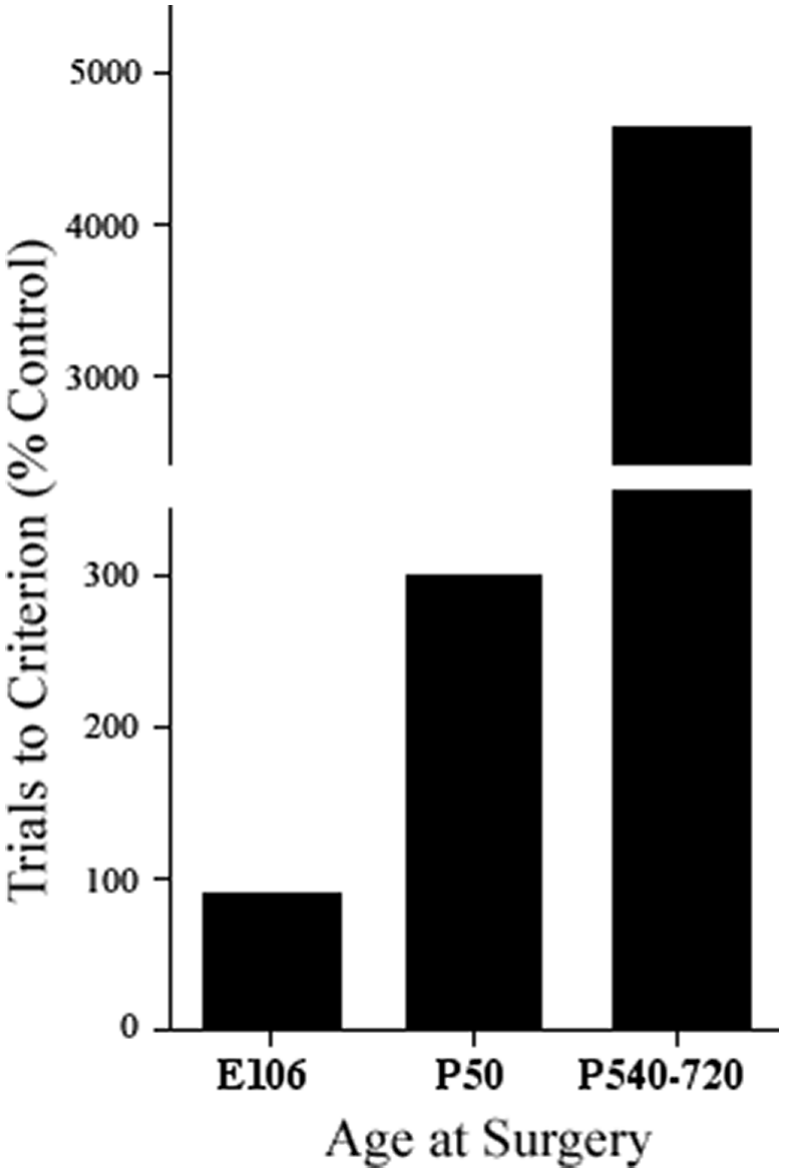
Performance on a test of delayed alternation by a prenatally prefrontal cortex operated monkey (E106), postnatally operated monkeys (P50), and adult operated monkeys (P540–720) (adapted from Goldman and Galkin, 1978).

One additional conclusion from Pat’s initial studies was that there was no evidence from the monkey studies that anomalous connections underlay functional sparing from early lesions. However, there was evidence from studies in both cats with infant prefrontal lesions (e.g., Villablanca et al., 1978, 1993) and rats with infant medial frontal lesions (e.g., Kolb and Nonneman, 1976, 1978) showing that not only was there sparing of function after early lesions but that this was correlated with morphological remodeling (Kolb et al., 1994). A fundamental difference between monkeys and cats and rats is that monkeys are developmentally much older than cats or rats. This suggested that there was a critical period in brain development, roughly when neurogenesis was just complete, when the brain could compensate for the injury. We might predict, therefore, that if monkeys were given dlPFC lesions prenatally, corresponding to the developmental age of cats and/or rats, they might not show delayed-response deficits, regardless of when they are tested behaviorally. Indeed, Pat showed this to be the case (Goldman and Galkin, 1978) (see Figure 2). Furthermore, she showed anatomical compensation in her fetal-operated monkeys, confirming that anomalous brain development can support the sparing of function after early injury in monkeys, as much as had been reported in cats and rats.

In sum, the importance of Pat’s studies of early prefrontal lesions in monkeys cannot be underestimated. Prior to her studies, the Kennard Doctrine was largely accepted. Pat’s studies revealed that although her fetal frontal lesion study provides some support for the Kennard Doctrine, the story is far more complicated and interesting. The effects of early cortical lesions are modulated by precise developmental age at injury and precise age at behavioral assessment. In a sense, monkeys with early lesions can “grow into behavioral deficits,” which is a finding with important clinical and legal implications. On a personal note, I was doing parallel studies on early brain injury in rats beginning in the early 1970s and continuing for the next 40^+^ years, and Pat’s monkey studies profoundly influenced my own thinking and research.

## Studies of prefrontal cortex anatomy

Although today there may be a tendency to focus on the findings of non-invasive imaging to study the functions of brain regions, such current studies have been guided by neuroanatomical studies dating back to the early 1900s when anatomists such as Brodmann identified multiple regions within the frontal lobe. However, it was not until the late 1940s that techniques began to be developed that allowed researchers to identify connections between different regions. For example, the classic definition of the prefrontal cortex was based upon a study by Rose and Woolsey (1948) in which they showed that the mediodorsal nucleus of the thalamus (MD) of the rabbit, sheep, and cat projected to a region at the front of the cerebral hemispheres. Given that primary sensory and motor regions are defined by their thalamic connections, Rose and Woolsey suggested that the MD projection defined the prefrontal cortex. Following up on Rose and Woolsey’s findings, later studies showed that the MD projection could be used to define the prefrontal region of other species such as monkeys, dogs, cats, and rats (e.g., Akert, 1964; Leonard, 1969). However, as new and more sensitive techniques were developed, things proved to be much more complicated. Pat et al. used more sensitive techniques to re-examine the MD-PFC relationship and showed that a revision was needed because MD projected outside prefrontal regions defined by cytoarchitectonics and the other thalamic regions also project to the prefrontal regions (Goldman-Rakic and Porrino, 1985; Giguere and Goldman-Rakic, 1988). This study was seminal as it provided a basis for re-examining the thalamic connectivity of the prefrontal cortex across mammalian species, and it became clear that there were significant species differences in the organization, and presumably details of function, across species such as monkeys and rats (e.g., Preuss, 1995).

**Figure 3:**
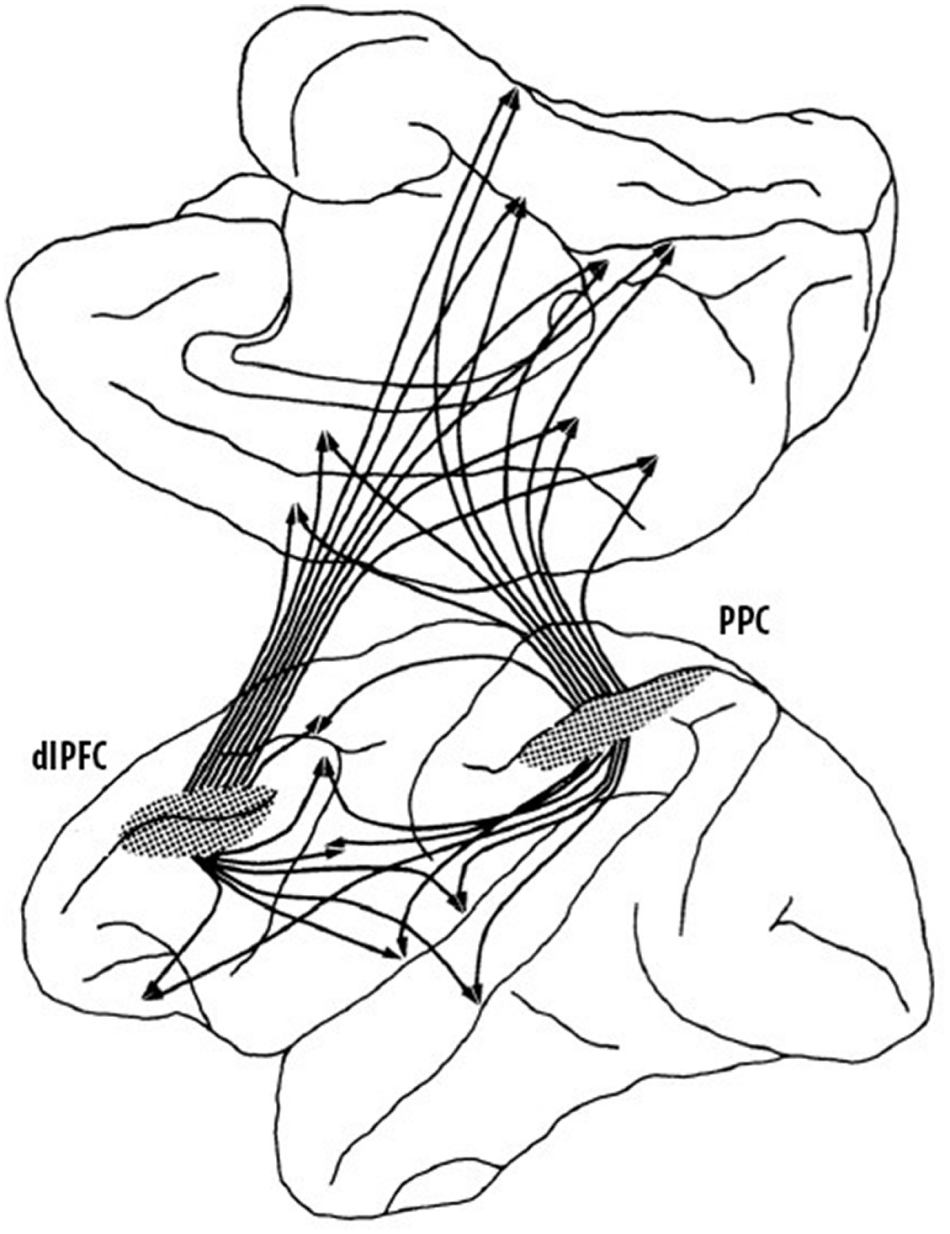
Summary of dorsolateral prefrontal (dlPFC)—posterior parietal (PPC) cortical regions. These two cortical regions are highly interconnected (not shown) and have similar connections to widespread regions of the cortex as well as subcortical connections that are not shown (adapted from Selemon and Goldman-Rakic, 1988) Copyright 1988 Society for Neuroscience.

It was not only thalamo-prefrontal connectivity that proved important in understanding the organization and function of the prefrontal cortex but it was also the study of cortico-cortical connections that radically changed the understanding of the role of the prefrontal cortex in cognitive functions. Nauta (1964) had begun to explore efferent connections of the prefrontal cortex of the rhesus monkey, but he wrote that “this report can be no more than a preliminary note.” He was implying that there was a lot more to learn as his study had only scratched the surface. In the 1970s, Pat collaborated with Nauta to learn his new tracing techniques and one of their first findings was the first evidence that cortical–cortical connections of the dlPFC had a columnar organization much like what had previously been described for the visual cortex (Goldman and Nauta, 1977). This was a game changer because it implied that columnar organization of cortico-cortical connections was likely a general characteristic of cortical organization and not simply a characteristic of the visual cortex. Furthermore, in a later study, Pat showed that the columns were present before birth (Schwartz and Goldman-Rakic, 1991), which meant that there was a genetic basis for the columnar organization and thus not experience-dependent. Another critical finding was that the dlPFC and the posterior parietal cortex (Brodmann’s area 7; von Economo’s area PE) not only had reciprocal connections to one another but that they also *both* projected in a columnar fashion to a wide range of cortical regions including the OFC, premotor cortex, anterior cingulate cortex, posterior cingulate cortex, area 19 of visual cortex, the superior temporal sulcus, parahippocampal cortex, insula, and retrosplenial cortex (Selemon and Goldman-Rakic, 1988). This complex pattern of dlPFC-posterior parietal connections forms the basis of an extensive network, summarized in Figure 3, which roughly corresponds to the default network identified in later non-invasive imaging studies in humans. In parallel, Pat et al. also described an extensive pattern of aligned connections from the dlPFC, OFC, temporal, and posterior parietal regions to adjacent regions of the striatum, suggesting a fundamental anatomic property of cortical-subcortical connections in which different cortical regions projected to independent striatal compartments, rather than being overlapping as they were thought to be (Selemon and Goldman-Rakic, 1985).

Taken together, Pat’s anatomical studies radically changed our understanding of how the prefrontal cortex functionally interacts with other cortical regions. This is important not only for understanding the effects of lesions on cognitive functions in human and non-human brains but also for providing a schema for the explosion of later studies using MRI-related techniques.

## Studies of working memory

In 1890, William James drew a distinction between memories that endure only briefly and memories that are longer term. This distinction was largely ignored until Donald Broadbent specifically postulated separate short- and long-term memory systems in 1958 although he had no model for how the brain did this. Short-term memory, also known as *working memory* or *temporal memory,* is a neural record of recent events and their order. Consider the delayed-response test that was first used by Jacobsen to study his non-human primates with frontal lobe lesions. There is a cue showing where a reward can be found, but it is then hidden from view by an opaque screen. After a delay of a few seconds, the screen is removed, and the subject can choose the correct location from two or more choices. The only solution is to recall the information from the ongoing trial because the correct location moves from trial to trial.

Beginning with Jacobsen’s first demonstration of a delayed-response deficit in two chimpanzees with large frontal lobe lesions, the delayed-response deficit has been associated with prefrontal injury (Goldman-Rakic, 1987) and has been shown in a range of laboratory species including monkeys, cats, dogs, and rats as well as in humans.

As noted earlier, Jacobsen’s findings went largely unstudied for over 20 years until several laboratories began to follow up on Jacobsen’s results (e.g., Rosvold et al., 1961; Gross and Weiskrantz, 1962). Pat’s first contribution to this area was her 1970 article with rosvold Goldman and Rosvold (1970) in which they showed that two regions in the dlPFC, one in the principal sulcus and one in the nearby arcuate sulcus, played separate roles in spatial memory. Removal of the principal sulcus impaired performance on a spatial task with delay, whereas removal of the arcuate sulcus impaired performance on a spatial task without delay. This led them, along with others, to suggest that the cortex in the principal sulcus was concerned with a form of spatial memory. A follow-up study by Brozoski et al. (1979) made a remarkable novel finding that depletion of dopamine in the principal sulcus produced a spatial delayed-response deficit that was nearly as severe as the effect of surgical ablation. Furthermore, the behavioral deficit could be reversed by dopamine agonists such as L-dopa. The role of dopamine is discussed below.

Electrophysiological studies using monkeys in the 1970s first showed that prefrontal neurons were highly active during the delay, ending with the response (e.g., Fuster and Alexander, 1971; Kubota and Niki, 1971). This finding suggested that the prefrontal neuronal activity was the basis of the working memory. If the neurons stopped firing before the delay was complete, the performance fell to chance because the memory was lost.

Pat et al. built on the 1971 findings and discovered that visual working memory was far more complex than it appeared. First, they showed that prefrontal neurons have “memory fields” as they respond to a visual target in one or a few locations in the visual field (Funahashi et al., 1989, 1990). In their task, a monkey was required to fixate on a central spot of light while a light flashed somewhere in the visual field. After a delay of 3 s, they were then required to make a saccadic eye movement to one of four or eight locations where the visual cue might have been presented. Neurons were recorded from the dlPFC and approximately 50% had task-related activity and approximately 20% had phasic visual responses to the onset of the visual cues. Most of the visual responses occurred only for location cues in a restricted portion of the visual field, mostly to the contralateral visual field although some did respond to the ipsilateral field or along the visual meridian. The activity of the neurons was the basis of the working memory as mentioned in the 1971 studies.

Of course, memory is not the action of a single neuron but rather many neurons working together. In her influential review of the cellular basis of working memory (Goldman-Rakic, 1995), which currently (December 2023) has over 3,000 citations, Pat hypothesized that pyramidal cells with similar “best spatial locations” are interconnected in deep layer III, much like the primary visual cortex neurons with similar orientation specificity that are linked together. Her idea was that extensive recurrent excitation among these neurons was the basis for the persistent firing, whereas lateral inhibition would refine the spatial tuning of the neurons.

**Figure 4:**
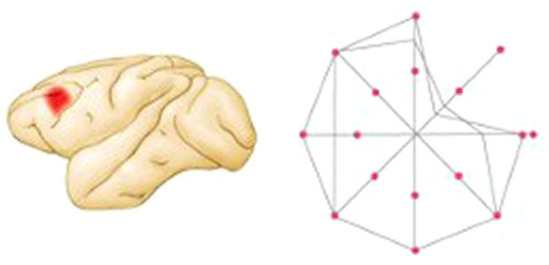
Summary of performance of a monkey with a left dlPFC lesion on a visual delayed-response task in which the monkey is trained to fixate at a central point and then after a 3 s delay to move the eye to locate the place where a target light had flashed. Correct performance percentage is indicated by the relative positions of the lines along axes drawn through the central fixation point. The monkey performed poorly in one region of the visual field contralateral to the lesion (figure adapted from Kolb and Whishaw, 2020; based on data from Funahashi et al., 1989).

In a subsequent study, the authors made small dlPFC lesions and showed that in contrast to the pre-lesion performance in which the monkeys responded as in the earlier neurophysiological studies, the monkeys had disrupted performance to the spatial cues in a restricted region of the visual field contralateral to the lesioned hemisphere (see Figure 4) (Funahashi et al., 1993a,b). Thus, the monkeys had mnemonic “scotomas.” In other words, although the monkeys still had a working memory for the location of most flashes, there were small regions for which there was no memory. As in the typical delayed-response task, the effect of the lesions was delay-dependent: performance was rarely altered at the shortest (1.5 s) delay but became progressively worse as the delay period was lengthened. Of special interest was the finding that the working memory mechanisms were lateralized: memories for visual-spatial coordinates in each hemifield were processed primarily in the contralateral dlPFC. This was a remarkable finding that changed the way the field thought about both working memory and prefrontal organization. Working memory was not a general process of the dlPFC but was the result of multiple processes originating from specific populations of neurons.

In a subsequent study using the same task, Pat’s group made another remarkable finding related to the role of dopamine in spatial memory. It was known from many studies (e.g., Berger et al., 1988; Williams and Goldman-Rakic, 1993) that the PFC receives a dense dopamine innervation. To further examine the role of the dopamine input into the dlPFC on the working memory, they injected dopamine antagonists locally into the dlPFC and identified specific target locations that induced deficits in task performance (Sawaguchi and Goldman-Rakic, 1994). As with the earlier lesion study with the memory fields task, the effects were most often in the field contralateral to the injection site and the degree of deficit was sensitive to the duration of delay. In addition, the deficit was dose-dependent: Higher doses induced larger deficits. By varying the type of dopamine antagonist, they were also able to show that it was only the D1—dopamine receptors that induced the deficits. The role of dopamine in short-term memory is important because many factors alter dopamine innervation to the prefrontal cortex, including Parkinson’s disease, schizophrenia, and aging.

**Figure 5:**
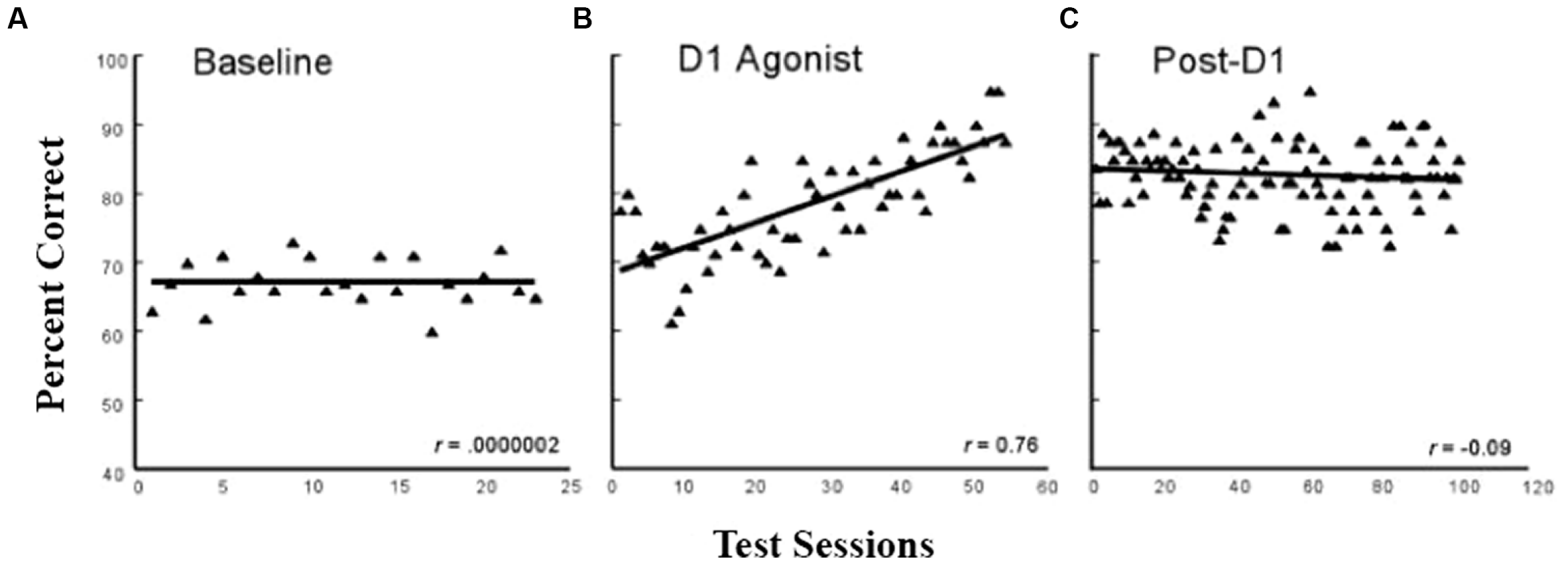
Delayed response performance across baseline, D1 agonist, and post-D1 agonist testing periods in aged monkeys. The filled triangles represent the averaged performance of four aged monkeys on a spatial working memory task for individual test sessions across the baseline. (adapted from Castner and Goldman-Rakic, 2004) Copyright 1988 Society for Neuroscience.

During the process of aging, a pronounced loss of dopamine function is associated with reduced working memory capacity (e.g., Arnsten et al., 1994). One logical question that Pat next asked was whether enhancing D1 receptor stimulation in dlPFC could enhance working memory in aged monkeys. Thus, Castner and Goldman-Rakic (2004) trained aged (20–30^+^ years of age) and young (7–10 years of age) monkeys on a standard delayed-response task. This was a lengthy study (3^+^ years) because the animals were first trained to 90% accuracy at a very short delay (1 s) and then gradually increased the delay to up to 10 s such that all monkeys were responding correctly at a stable 70% accuracy for a minimum of 20 consecutive test sessions. (There were 3–5 test sessions a week.) Once the monkeys reached this baseline level, they received intermittent treatment with a selective D1 agonist (ABT-431), and then, the monkeys were trained on the delayed-response task for 60 training sessions. As shown in Figure 5, the aged monkeys showed a significant improvement to almost 90% accuracy. After a drug washout period, the animals were retested for another 120 sessions and showed a persistent cognitive improvement as shown in Figure 5. The young monkeys showed no significant benefit of the treatment either during or post-D1 treatment. This study has obvious implications for the enhancement of cognitive functions in the growing human elderly population and provides a basis for drug development targeted to this group. To date, other than studies with dopamine agonists such as L-dopa for Parkinson’s patients, which cannot be used for normal aging owing to the side effects of the drug, there are no new pharmaceutical agents designed for the effects of “normal aging,” but this is obviously an important direction for the future.

## Schizophrenia and prefrontal cortex

It has been known for a long time that there is a relationship between dopamine and schizophrenia. The evidence supporting this relationship comes from many sources including postmortem studies showing an imbalance of dopamine and its metabolites in patients with schizophrenia, possibly resulting, in part, from excessive DA receptors in the midbrain and reduced DA receptors in the prefrontal cortex (Purves-Tyson et al., 2017; McCutcheon et al., 2020). In addition, there is considerable evidence showing that drugs that block the receptors for dopamine can reduce schizophrenic symptoms (Ceraso et al., 2020).

Given Pat’s research showing the role of dopamine in working memory (see above), Pat noted that the profound information-processing deficits in schizophrenia included a severe working memory deficiency (e.g., Goldman-Rakic, 1994; Goldman-Rakic and Selemon, 1997). People with deficits in working memory do not typically have amnesia, agnosia, or aphasia, and their sensory and motor capacities are normally within the normal range. Pat argued that the basic problem appears to be a deficit in developing a concept, idea, or schema, based in part on past experiences, in order to guide ongoing behavior. The challenge is to identify a neurobiological mechanism underlying the cognitive problems.

There are two general ways to approach this issue. One is to use non-invasive imaging such as positron emission tomography (PET) and functional MRI (fMRI) as participants engage in tasks such as working memory or other cognitive problems. Unfortunately, Pat passed away just as the use of these techniques was emerging in neuroscience, but she did do pioneering fMRI studies of working memory in both spatial and non-spatial working memory tasks (McCarthy et al., 1994, 1996) and schizophrenia (Driesen et al., 2008). To date, however, the imaging results have proven to be complex, in part because of the wide variation in research study results (Chatterjee and Chatterjee, 2023).

A second way to look for a neurobiological mechanism is to identify the neuropathology of schizophrenia using postmortem analyses, structural MRI, and CT scans. Given that Pat’s laboratory had identified anatomical evidence for powerful prefrontal regulation of posterior cortical areas (see Figure 1), it was a logical step for her to search for changes in the nature of the prefrontal-posterior cortical networks that could account for at least some of the cognitive deficits. There had been CT scan findings that schizophrenic patients have larger ventricles than controls (Johnstone et al., 1976), thus confirming the idea that brain changes were a part of the disease. Subsequent macroscopic studies confirmed this finding and showed decreases in both gray matter and brain volume (see a review by Bakshshi and Chance, 2015), but the key problem is in identifying microscopic changes that could be linked to behavioral symptoms. One of the most influential proposals was put forward by Selemon and Goldman-Rakic (1999) when they proposed the “reduced neuropil hypothesis” (see also Selemon et al., 1998). Based on postmortem examination of the frontal lobe of schizophrenic patients, they found reduced neuropil in dlPFC, which resulted in increased cell density owing to reduced dendrites, axons, and terminals. This was the first study to show that the dlPFC was altered in schizophrenia and the study has been influential, having been cited 1,130 times as of December 2023 (Google Scholar). Subsequent studies by others have replicated the reduced neuron density finding (e.g., Glantz and Lewis, 2000) and have also shown that reduced neuronal density in schizophrenia may be a general effect across much of the cortex, which makes sense given the extensive prefrontal cortical connections not only across the cortex but also across subcortical regions as well. Furthermore, given the heterogeneity of behavioral symptoms in schizophrenia, it is not surprising that subsequent studies have shown other microscopic changes in schizophrenia including changes in cell size and number, changes in microglia (see review by Bakshshi and Chance, 2015), and evidence that abnormalities in other transmitters, especially glutamate, are also involved (McCutcheon et al., 2020). More recently, using connectomic and predictive models on neuroimaging datasets including schizophrenic patients and healthy controls, Wang et al. (2023) have identified two patterns of dysconnectivity for cortico-cortical and cortico-striatal circuits, each associated with specific clinical symptoms. Nonetheless, Pat’s research was a beacon for others to follow as it was the first to show a cellular basis for the cognitive changes in schizophrenia.

## The lasting impact

It would be difficult to capture the total impact that Pat has had in over 300 scholarly articles (see also reviews by Arnsten, 2013, 2023). My goal here was to emphasize her crucial role in stimulating the study of the PFC at a time when the field was far more focused on the visual and hippocampal systems. What is remarkable is not that Pat had an impact, but that even 20 years after her death her impact remains so strong. One change in the past two decades is the advent of cognitive neuroscience, and the extensive use of neuroimaging techniques has shifted attention away from the use of chronic, focal lesion studies, which characterized Pat’s behavioral studies, and were the mainstay of neuroscience for decades in the study of both human and non-human brain function. A review by Vaidya et al. (2019) provides a re-examination of the role of lesion and anatomical studies in cognitive neuroscience, and they conclude that lesion and anatomical studies provide vital insights into brain function that cannot be achieved by correlational studies of brain activity. Even today, the integration of insights gained from lesion and anatomical studies, such as those by Pat et al., with results from other methods remains crucial for advancing neuroscience. The importance of Pat’s lesion and anatomical studies of the frontal lobe is reflected in Pat’s citation statistics. As of November 2023, Pat has over 100,000 citations and a h-factor of 158 as seen in Google Scholar. This includes nearly 15,000 citations in the past 5 years. To put this in perspective, most neuroscientists do not have 15,000 citations over their career, let alone 15–20 years after their passing. Just imagine what her impact would have been had she continued her research in the decades following her death.

However, Pat’s impact goes well beyond publication statistics. Pat began her research career at a time when there were very few women in neuroscience, and she provided a role model for women in neuroscience and science in general. It is interesting to note that of her eight articles published with co-authors at Yale that are cited in this review, six of the co-authors are women, which given the time that they were published is remarkable.

Finally, Pat’s research was driven by big questions that allowed a top-down strategy in which she used multiple methods to look for answers, rather than starting with a method and looking for a question or using only a single method. My emphasis in this review has been on her pioneering study using behavioral and anatomical studies that had a major role in bringing behavioral neuroscience to the study of the prefrontal cortex, not only in non-human primates but in other species as well. In 1970, when Pat published her first three articles on the prefrontal cortex, there were only a handful of publications on the prefrontal cortex of any species, including those in the Warren and Akert (1964) volume, but according to PubMed, in November 2023, there were close to 43,000 using monkeys to study PFC and an overall total of 68,000 that includes other species as well. Pat’s study was pioneering, and although she was not directly responsible for all these works, her pioneering study paved the way.

## Author contributions

BK: Writing – original draft.
